# Does salt stress constrain spatial distribution of dune building grasses *Ammophila arenaria* and *Elytrichia juncea* on the beach?

**DOI:** 10.1002/ece3.3244

**Published:** 2017-08-08

**Authors:** Marinka E. B. van Puijenbroek, Corry Teichmann, Noortje Meijdam, Imma Oliveras, Frank Berendse, Juul Limpens

**Affiliations:** ^1^ Plant Ecology and Nature Conservation Group (PEN) Wageningen University & Research Wageningen The Netherlands; ^2^ Environmental Change Institute School of Geography and the Environment University of Oxford Oxford UK

**Keywords:** *Ammophila arenaria*, biogeomorphology, ecophysiology, *Elytrigia juncea*, incipient dunes, soil salinity, The Netherlands

## Abstract

Rising sea levels threaten coastal safety by increasing the risk of flooding. Coastal dunes provide a natural form of coastal protection. Understanding drivers that constrain early development of dunes is necessary to assess whether dune development may keep pace with sea‐level rise. In this study, we explored to what extent salt stress experienced by dune building plant species constrains their spatial distribution at the Dutch sandy coast. We conducted a field transplantation experiment and a glasshouse experiment with two dune building grasses *Ammophila arenaria* and *Elytrigia juncea*. In the field, we measured salinity and monitored growth of transplanted grasses in four vegetation zones: (I) nonvegetated beach, (II) *E. juncea* occurring, (III) both species co‐occurring, and (IV) *A. arenaria* dominant. In the glasshouse, we subjected the two species to six soil salinity treatments, with and without salt spray. We monitored biomass, photosynthesis, leaf sodium, and nutrient concentrations over a growing season. The vegetation zones were weakly associated with summer soil salinity; zone I and II were significantly more saline than zones III and IV. *Ammophila arenaria* performed equally (zone II) or better (zones III, IV) than *E. juncea*, suggesting soil salinity did not limit species performance. Both species showed severe winter mortality. In the glasshouse, *A. arenaria* biomass decreased linearly with soil salinity, presumably as a result of osmotic stress. *Elytrigia juncea* showed a nonlinear response to soil salinity with an optimum at 0.75% soil salinity. Our findings suggest that soil salinity stress either takes place in winter, or that development of vegetated dunes is less sensitive to soil salinity than hitherto expected.

## 1 INTRODUCTION

Sea levels are predicted to rise with 26–82 cm in this century, due to climate change (IPCC, 2014). Rising sea levels may lead to higher frequency and intensity of flooding, emphasizing the need for flexible coastal protection (IPCC, 2014; KNMI & PBL, 2015). Coastal dunes provide such a flexible, natural form of coastal protection, while also providing other important ecosystem services such as freshwater supply, recreation, and biodiversity conservation (Everard, Jones, & Watts, 2010). Understanding the factors that constrain early dune development is essential to predict whether natural coastal protection can keep pace with the rising sea level.

Coastal dune formation is the result of vegetation growth and eolian processes (Hesp, 2002). Nonvegetated dunes can form by eolian transport of sand, but these dunes are transient and will disappear when the wind direction changes. Once vegetation established on the beach, it captures wind‐blown sand and forms an embryo dune (also known as an incipient dune; Hesp, 2002; Maun, 2009; Zarnetske et al., 2012). These vegetated embryo dunes may grow into foredunes that are known for their coastal protection function (Maun, 2009). As vegetation plays a key role in capturing and retaining sand, the position and rate of dune development on the beach are constrained by vegetation establishment and growth (Keijsers, De Groot, & Riksen, 2015; Zarnetske et al., 2012).

Vegetation growth on the beach is limited by the harsh environmental conditions (Maun, 2009), including high salinity (Maun, 1994). Plants experience the saline conditions both aboveground by salt spray and belowground by salt concentration (soil salinity). Both salt spray and soil salinity decrease from beach toward dunes (Gooding, 1947). Salt spray on the beach strongly depends on wind speed and precipitation (Boyce, 1954), while soil salinity is influenced by inundation by seawater, saline groundwater, salt spray, precipitation, moisture content, and soil texture (Martin, 1959). Salt spray and soil salinity can disrupt plant–water relations, promote tissue necrosis and leaf loss, reduce photosynthesis, and reduce growth in exposed plants (Boyce, 1954; Breckle, 2002; Munns & Termaat, 1986). Consequently, these factors can have a great impact on the distribution of plant species, and thus potential dune formation, on the beach.

In western Europe, the main two dune building species are *Ammophila arenaria* (L.) Link and *Elytrigia juncea* (Simonet & Guin). *Ammophila arenaria* has been introduced in many countries, because of its excellent dune building capabilities (Konlechner, Hilton, & Orlovich, 2013). *Ammophila arenaria* creates higher, more hummocky peaked dunes (Hesp, 2002), which can easily withstand flooding (Seabloom, Ruggiero, Hacker, Mull, & Zarnetske, 2013). *Elytrigia juncea* creates lower broader dunes, and the distribution is more restricted to Europe. *Elytrigia juncea* usually grows closer to the sea than *A. arenaria* (Bakker, 1976). It is generally assumed that dune building starts with *E. juncea* and, once when a freshwater lens is formed, *A. arenaria* plants establish and over time outcompete *E. juncea* (Westhoff, Bakker, van Leeuwen, & van der Voo, 1970). The order in which the grass species occur on the beach corresponds with their salinity tolerance investigated under controlled conditions (Rozema, Bijwaard, Prast, & Broekman, 1985; Sykes & Wilson, 1988, 1989; Data S1), suggesting dune building is constrained by soil salinity. However, studies that actually measured both vegetation distribution and environmental conditions in the field (de Jong, 1979; Maun, 2009) conclude that soil salinity on the beach is lower than generally assumed and is unlikely to limit plant growth on the beach. It is yet unclear what explains the discrepancy between spatial plant distribution on the beach, salinity‐tolerance ranges measured in short‐term physiological studies, and the actual salinity measured on the beach. Did the physiological studies (Rozema et al., 1985; Sykes & Wilson, 1988, 1989) underestimate the cumulative effect of salt stress (Munns, 2002) due to their short duration (4–10 weeks) or can it be that interactive effects of salt spray and soil salinity explain why plants did not occur under the relatively low soil salinity measured in the field?

In this study, we try to bridge the gap between field and glasshouse studies by conducting a field experiment with *A. arenaria* and *E. juncea* transplanted into different vegetation zones and by comparing the field response with a full factorial glasshouse experiment where we subjected the two species to different soil salinities with and without salt spray. Specifically, we attempted to answer the following research questions: (i) What are the interactive effects of salt spray and soil salinity stress on growth of *E. juncea* and *A. arenaria*? (ii) Which physiological mechanisms (osmotic stress, ionic stress, and nutrient limitation) can explain their biomass response? (iii) Does their response to salt spray and soil salinity explain the growth of *A. arenaria* and *E. juncea* in the field?

## 2 METHODS

### 2.1 Field transplantation experiment

We conducted a field experiment to assess the plant growth of *A. arenaria* and *Elytrigia juncea* along five transects from beach to dune (Data S2) on the Hors on Texel, a barrier island in the Netherlands (coordinates: 52°59′51.97″N, 4°44′04.83″E). The Hors is a wide dissipative beach with much hydrodynamic reworking of the sand, which results in a high transport potential and opportunity for dunes to develop. Due to relatively storm free periods, many dunes have been able to develop on the Hors in the last 20 years. Within each transect, we selected four locations representing different stages of dune development, zone (I) the nonvegetated zone above the mean high water line, 0.78–1.1 m NAP (NAP refers to Amsterdam Ordnance Datum, which is equal to mean sea level near Amsterdam); (II) zone with *E. juncea* occurring, 1.17–1.19 m NAP; (III) zone with both species co‐occurring, 1.42–1.94 m NAP; and (IV) zone where *A. arenaria* is dominant, 2.06–3.17 m NAP. At each location, we established six plots of 50 × 50 cm. The minimum distance between the plots was 2 m. Three treatments were randomly assigned to the plots: monoculture of *A. arenaria,* monoculture of *E. juncea*, and mixed culture of *A. arenaria* and *E. juncea*. In each plot, we planted 20 plants; in the mixed culture, we planted 10 plants of each species. The plants, consisting of one shoot, were collected from the same site and stored outside in plastic bags with moist sand for a maximum of 2 weeks until planting.

After planting, we standardize the leaf height between species and plots by clipping the leaves until the leaves were 3 cm long. We established the experiment in the end of March 2014. We measured the number of leaves for a fixed subplot of 30 × 30 cm within each plot in May–October 2014, and August 2015.

### 2.2 Soil salinity measurements in the field

We measured the soil salinities at the locations where we established our field experiment. At each location, we took soil samples from five depths (5, 10, 25 and 50 cm). The samples were taken back to the laboratory and dried at 105°C. The dried soil samples were diluted on a 1:5 mass basis with distilled water. The electrical conductivity of this solution was measured and multiplied with a factor 17 to derive the EC at saturated conditions (ECe) (Shaw, 1994). When there was groundwater at the sampling depth, we measured groundwater salinity directly in the field with the same instrument as used in the laboratory. The groundwater depth ranged between 44 cm and >75 cm below beach surface, depending on location and transect. The measurements were performed on 12, 13, and 14 August 2015. While 12 and 13 August were dry, there was precipitation (15 mm) in the early morning of the 14 August which slightly reduced the soil salinity of one of the five transects, increasing the error bars per location.

To explore whether the soil salinity on Texel is comparable to other beaches along the Dutch coast, we complemented our data with soil salinity measurements on two additional beaches: the Hondsbossche Duinen (HD), in North Holland (coordinates: 52°44′34.31″N, 4°38′33.14″E; date: September 2015) and on Terschelling, another barrier island (coordinates: 53°24′30.31″N, 5°17′29.25″E, measured in June, August, and November 2015). Both beaches are dissipative beaches; however, they have a smaller beach compared to the Hors. The HD is an artificial created mega‐nourishment and has the smallest beach width, whereas the beach on Terschelling has a much wider beach width. On HD, we measured soil salinity at the upper beach and dune foot, 1.9–2.5 m NAP, and on Terschelling, we measured at the upper beach, 1.9–2.3 m NAP.

The summer of 2014 was warmer and wetter compared to previous years, ideal conditions for plant growth (van Puijenbroek, Limpens, et al., 2017). The average temperature in June and July was 17.40°C, and the precipitation over the growing season was 361 mm (KNMI, 2015). Over the winter, there were two major storms and highest water level was 248 cm NAP, and this water level occurs once every 2 years. This water level is higher than most of our plot locations: only three plot locations in zone IV had a higher elevation. However, it is likely that also these locations became inundated due to wave run‐up, as suggested by the position of the tidemark. The storm eroded part of the beach, the beach width decreased, but beach elevation did not change.

### 2.3 Glasshouse experiment

#### 2.3.1 Plant material

We collected 600 rhizomes equally divided over both *A. arenaria* and *E. juncea*, from the vicinity of our field transplantation experiment on the Hors, Texel. The rhizomes were stored in plastic bags with moist sand in a fridge (*c*. 4°C) for 3 weeks until planting. Just before planting, we standardized the rhizomes by cutting all of them to similar length (20 cm), and it was not possible to standardize the number of nodes on each rhizome. The range in node number was for *A. arenaria* 6–11 and for *E. juncea* 8–24. The rhizomes were planted in 196 experimental pots (10 L volume) filled with 14 kg soil, which consisted of a mixture of (calcareous) sandy river soil and organic matter (3:1 volume mixture) and one liter of water. Three rhizomes of one species were planted in each experimental pot, about 5 cm below the soil surface. All pots were watered every week to keep the soil moisture content constant, and no additional nutrients were provided during this initial phase. Shoots emerged from the rhizomes 1–4 weeks after the planting. Four weeks after the planting of the rhizomes, treatments were randomly assigned to all pots where tillers had developed. We ended up with 192 pots for the main experiment (see experimental design below), leaving four pots with living tillers to verify the experimental treatments. The glasshouse climate for both preparation phase and experiment was set to 20°C at day and 15°C at night, standard humidity (about 50%). Natural light was supplemented by SON‐T 400W lamps to guarantee 16 hr day length.

#### 2.3.2 Experimental design

A total of 192 experimental pots were used in this experiment with a full factorial design of two factors: soil salinity (six different levels) and salt spray (with/without). For each treatment, we had eight replicates, which were distributed over eight replicate blocks. Within each block, the treatments were repeated for each species: *A. arenaria* and *E. juncea*. The position of the experimental blocks was randomized three times during the experiment to control for potential variation in light conditions within the glasshouse. For the salt spray treatment, the plants were initially sprayed five times from all sides at 70 cm distance with either distilled water or water with 3.5% NaCl concentration (Sykes & Wilson, 1988). After 14 weeks, we increased the spraying treatment by spraying ten times from all sides, to ensure that all leaves were sprayed. While spraying, waterproof cardboard was used to shield the other pots from the spraying. For the soil salinity treatment, six different saline solutions were prepared with 0%, 0.25%, 0.5%, 0.75%, 1.0%, and 1.5% salt concentration (corresponding to 0, 42.8, 85.6, 128, 171, 214 mmol/L NaCl, and 0.28, 6.0, 11.1, 16.2, 20.2, 33.90 mS/cm EC). The soil salinity treatments were based on the range of soil salinities we found in the field. To ensure, there was no effect of salt spray on the soil salinity we applied once every week first the salt spray treatment and then the soil salinity treatment. As the provided salt can accumulate in the soil, excess saline solution was supplied to the experimental pots to a set weight (16 kg). At this pot weight, about one‐third of the saline solution drained from the pots, preventing accumulation of salt at concentrations higher than the treatment (Poorter, Fiorani, Stitt, Schurr, & Finck, 2012; Sykes & Wilson, 1989). The saline solution was directly applied to the soil, to prevent a change in the salt spray on the leaves. Nutrients were added to the different saline solutions in the form of 2.5% Hoagland's solution, to ensure sufficient nutrients for plant growth. This low amount of nutrients represents the field conditions, as dunes are very nutrient poor (Maun, 2009). The plants were harvested after 25 weeks of the start of the treatments, which is more or less similar to the length of the growing season of the two dune building species.

#### 2.3.3 Plant growth

Plant growth was measured by counting the number of shoots, leaves (alive, dead), and the height of longest leaf for each experimental pot. Shoots were defined as an individual stem with leaves. Leaves were considered dead when they had no green tissue left. All variables were measured weekly during the first 12 weeks of the experiment and again during week 18 of the experiment. For nine of the 192 pots, all plants died during the experiment, all corresponding to *A. arenaria*. No pots with *E. juncea* experienced mortality; however, one experimental pot was planted erroneously with *A. arenaria* and was excluded from the analysis.

We harvested the experiment per block by collecting the whole plant after which we divided it into two fractions: the shoot (including both dead and alive leaves) and root biomass. The roots were carefully separated from the soil by gently rinsing them with flowing tap water. Biomass of both fractions was determined after drying the material at 40°C for 3 days.

#### 2.3.4 Measurements of gas exchange and stomatal conductance

We measured CO_2_ gas exchange and stomatal conductance to explore the mechanisms behind the biomass response. From week 21 to week 24 (May 1–21, 2015), we measured the leaf photosynthesis (CO_2_ net exchange) with a cross‐calibrated LI‐6400 portable photosynthesis system (LI‐Cor, Inc, Lincoln, NE, USA) from single leaves of all plants in four randomly selected blocks. The CO_2_ net exchange (Asat) was measured under ambient CO_2_ concentrations of 400 ppm and photosynthetically active radiation (PAR) flux density at or near 2,000 μmol m^−2^ s^−1^. Measurements were made from 08:30 to 12:00 hr during the day (CET time) to minimize the risk of declines in gas exchange rate as a result of stomatal closure, source–sink inhibition, or other causes during the afternoon (Pérez‐Harguindeguy et al. 2013).

The CO_2_ net exchange (Asat) and stomatal conductance were calculated with the following equations from von Caemmerer and Farquhar (1981).

1. Asat = (*F*(C_r_ – C_s_)/100S) – C_s_E
2. *g*_sw_ = 1/((1/*g*_tw_)–(*k*_ƒ_/*g*_bw_))

Asat is the photosynthesis in μmol m^−2^ s^−1^, *F* molar flow rate of air (μmol s^−1^), C_r_ and C_s_ are the sample and reference CO_2_ concentrations (μmol CO_2_ mol air^−1^), S is leaf area (cm^−2^), and E is the transpiration (mol H_2_O m^−2^ s^−1^). g_sw_ is the stomatal conductance in mol H_2_O m^−2^ s^−1^, *g*_tw_ is the total conductance (mol H_2_O m^−2^ s^−1^), *g*_bw_ the boundary layer conductance (mol H_2_O m^−2^ s^−1^), and *k*_ƒ_ is calculated by *k*_ƒ_ = (*K* ^2^ + 1)/(*K* + 1)^2^, where *K* is the stomatal ratio (estimate of the ratio of stomatal conductance of one side to the leaf to the other side).

Water use efficiency (WUE) was calculated as the ratio between the net CO_2_ exchange and the stomatal conductance. During the harvest, we measured the specific leaf area (SLA), for the four blocks where we measured the single leaf gas exchange. The SLA was measured by scanning five fresh undamaged leaves with a leaf scanner (Li‐3100 Area Meter) and weighing the dried leaves (dried at 40°). For each of these five leaves, we measured the leaf thickness.

#### 2.3.5 Plant chemical analyses

We measured the concentrations of nitrogen, phosphor, potassium (K), and sodium (Na) in the harvested shoot biomass of all plants in a subset of four randomly selected blocks. The concentrations of plant nutrients N, P, and K were measured to explore whether nutrient limitation could explain the plant biomass at higher soil salinity (Colmer & Flowers, 2008; Rozema, van Manen, Vugts, & Leusink, 1983). Concentrations of Na were measured to explore whether ionic stress played a role in explaining the treatment effect (Munns & Termaat, 1986). The harvested shoot biomass, which includes dead and alive biomass, was first gently rinsed with distilled water to remove any residual salt spray. The dried shoot material (70°C) was pulverized and digested with H_2_SO_4_, salicylic acid, H_2_O_2_, and selenium. Subsequently N and P concentrations were measured colorimetrically using a continuous flow analyser (SKALAR SAN plus system, The Netherlands). K and Na were measured by flame atomic emission spectroscopy (AES) (Walinga, Vark, Houba, & van der Lee, 1989).

#### 2.3.6 Soil and leaf salinity in experimental pots

To verify that soil salinity at the end of the experiment still matched the treatments, we collected soil samples from the experimental pots of four randomly selected blocks and measured the electrical conductivity (EC) and calculated the electrical conductivity at saturated conditions (ECe), using the same methods as for the field samples.

To test how the salt spray treatment affected leaf salinity, we used four planted test pots, which we once sprayed with water which contained 3.5% NaCl. The plants in these test pots were harvested, and the leaves were washed with 500 ml distilled water. The difference between the EC of the distilled water before and after the plants were washed was used to calculate the EC on the leaves by correcting it for leaf area. The EC on the leaves after spraying once was 131.1 μS/cm. This value is higher than freshwater levels, and as we did not wash the leaves, the salinity did accumulate over time.

### 2.4 Statistical analysis

#### 2.4.1 Field experiment

Data from the two subreplicates per location in the field were averaged, to avoid pseudoreplication. We analyzed the number of living leaves with a linear mixed model, where we account for the repeated measure by using plot number as a random intercept, with culture (mono or mixed), species, zone, and month as explanatory variables. We calculated the Chi‐squared values with an ANOVA type III SS (Fox & Weisberg, 2011), as it is robust for unequal sample sizes (Quinn & Keough, 2006). We corrected for the number of plants that were planted at the start of the experiment. The unequal sample sizes were a result of anthropogenic disturbance. All plants in the nonvegetated zone (zone I) were pulled out of the plots shortly after the start from the field experiment, preventing inclusion in our analyses. Two additional plots in the zone with only *E. juncea* (zone II) were destroyed in September 2014. We excluded these plots from this time point onward.

#### 2.4.2 Glasshouse experiment

For the glasshouse experiment, the numbers of living leaves, tillers, and maximum plant height were analyzed with a generalized linear model with a negative binominal distribution, and a normal distribution for maximum plant height (Quinn & Keough, 2006). We used species, time, soil salinity, and salt spray treatment as explanatory variables. The total biomass, shoot biomass, root biomass, and shoot to root ratio were analyzed with an ANOVA and with species, soil salinity, and salt spray treatments as explanatory variables. Between the different treatments, significant differences were calculated using the Tukey HSD test (Hothorn, Bretz, & Westfall, 2008).

The net CO_2_ exchange and stomatal conductance were analyzed with an ANOVA type III. Sample sizes ranged between one and six per treatment because we discarded replicates with negative intercellular CO_2_ concentrations from the analyses. We used species, soil salinity, and salt spray treatments as explanatory variables. The N, P, Na, and K concentrations in the leaves were analyzed with an ANOVA, and species, salinity, and salt spray treatment were used as explanatory variables.

We did not find any significant difference between the different blocks of the glasshouse experiment. The normality of the data and homogeneity of variance were checked graphically. Variable deviating from normality was transformed with a natural logarithm (stomatal conductance, WUE, Na concentrations, number of living leaves in the field) or a square root (field soil salinity and the salinity in the pots) before analysis. To facilitate interpretation, the figures are based on nontransformed data. All statistical analyses were performed with the program R 3.1 (R Core Team, 2016).

## 3 RESULTS

### 3.1 Soil salinity in the field

**Figure 1:**
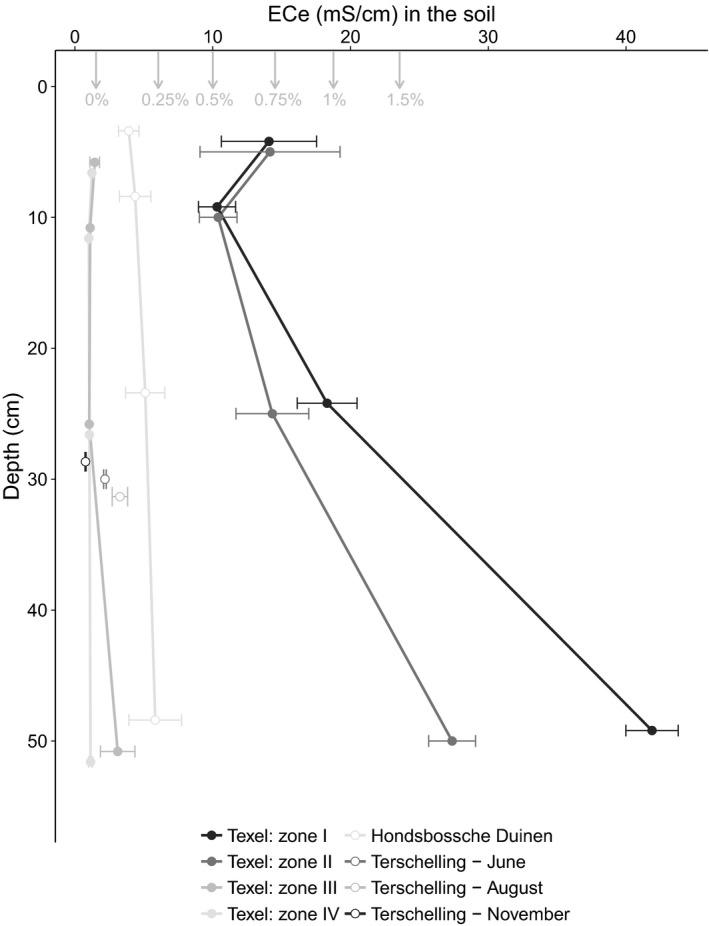
The ECe (Electrical conductivity at saturated soil) for different depths in the soil. Closed circles indicate the ECe at different zones at the Hors, Texel. Open circles indicate the ECe at beaches on the Hondsbossche Duinen (HD) and on Terschelling. Points show the mean and the error bars and the standard error. The arrows and the percentages show the ECe value of the specific soil salinity treatment. The EC of the seawater is 50 mS/cm. Zone I is the nonvegetated zone, zone II is the zone with only *Elytrichia juncea* occurring, zone III is the zone with both *E. juncea* and *Ammophila arenaria,* and in zone IV,* A. arenaria* is dominant

On Texel, the salinity of both groundwater and soil increased with proximity to the sea (*F*_89,5_ = 339.75, *p* < .001), but was only weakly related to vegetation zones (Figure 1). Zones I and II were saltier than zones III and IV. The salinity of the groundwater was 32.74 ± 6.12 mS/cm (means ± SE) in zone I and 5.63 ± 1.51 mS/cm in zone III (*F*_11,5_ = 19.96, *p* > .001). Soil salinity increased significantly with depth in zones I and II (*F*_89,5_ = 9.47, *p* < .001), whereas soil salinity was hardly affected by depth in zones III and IV (Figure 1). Soil salinity on Texel was significantly higher than on the narrower beaches of the HPZ or Terschelling, irrespective of vegetation zone.

### 3.2 Plant growth in field experiment

**Figure 2:**
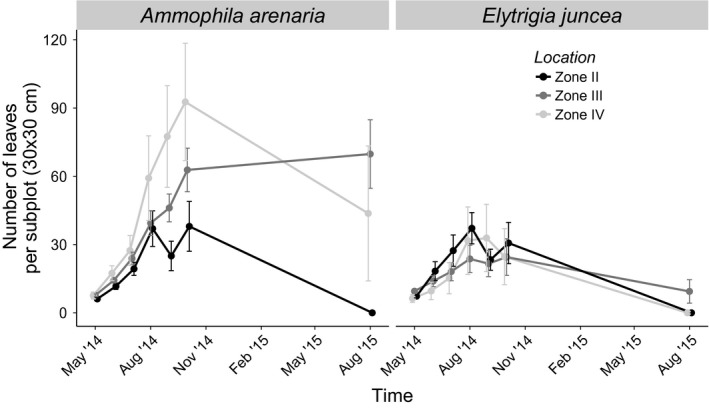
The number of living leaves for *Ammophila arenaria* and *Elytrichia juncea* per subplot of 30 × 30 cm within a plot at the different zones at the Hors, Texel, over a period of 15 months. The points are means, and the error bars are standard errors. Zone II is the zone with only *E. juncea* occurring, zone III is the zone with both *E. juncea* and *A. arenaria,* and in zone IV,* A. arenaria* is dominant

Plant growth depended on time of year, species, and zone. The number of leaves increased linearly after planting, levelled off at the end of the 2014 growing season, and declined over 2015, irrespective of zone and species. Species did show a different growth pattern over the zones (Figure 2), leading to a significant zone*species interaction(zone*species: *F*_2,338_=11.06, *p* < .004). *Ammophila arenaria* generally performed least in zone II, producing fewer leaves over summer and regrowing less after winter than in zones III and IV. *Elytrigia juncea* did not show a clear growth response to zonation: *E. juncea* performed equally well in all zones over summer, but only survived in zone III. There was no significant difference between the mixed and monoculture plots (mono vs. mixed *A. arenaria*: 1.87 ± 0.020 vs. 1.60 ± 0.017 leaves/planted plant, *E. juncea*: 0.91 ± 0.0095 vs. 0.60 ± 0.0064 leaves/planted plant, species * mono/mixed culture: *F*_1,338_=1.43, *p* = .23, Data S3).

### 3.3 Glasshouse experiment

The treatments resulted in the desired soil salinity concentrations (Data S4), irrespective of salt spray (*F*_77,1_ = 0.12, *p* = .73) and species (*F*_77,1_ = 0.25, *p* = .62). Soil salinity concentrations differed significantly between treatments, with soil salinity measured in the pots corresponding to the soil salinity treatment (*F*_77,5_ = 91.19, *p* < .001). Soil salinity in the treatments was within the same order of magnitude as the salinity measured in the field (Figure 1). The 0% salinity treatment corresponded to the soil salinity measured in zones III and IV (Figure 1), whereas the soil salinity for the 0.5%–1% soil salinity treatments corresponded to the salinity measured between 5 and 25 cm depth for zones I and II. The highest soil salinity treatment (1.5%) was found only deeper in the soil (>25 cm) in zones I and II.

### 3.4 Impacts on plant growth and biomass

Salt spray had neutral to positive effects on species performance. Salt spray significantly increase the number of leaves and tillers and the maximum plant height for *A. arenaria* (Table 1), but did not affect shoot, root, total biomass, and fraction of dead leaves of *A. arenaria* and *E. juncea* (Table 2). The total biomass for *A. arenaria* was 4.71 ± 0.42 g/pot without salt spray and 5.21 ± 0.45 g/pot with salt spray, whereas it was 8.28 ± 0.40 g/pot and 8.19 ± 0.41 for *E. juncea*. Salt spray interacted with soil salinity resulting in higher number of leaves for the lowest soil salinities (0%, 0.25%) for both species: In week 18, the number of leaves of *A. arenaria* for the 0% soil salinity treatment was 2.38 ± 0.42 without salt spray and 3.25 ± 0.31 with salt spray. Salt spray did not affect plant mortality: Of the nine pots where all plants died, five received salt spray. We found no significant interaction effect between salt spray treatment and soil salinity treatment for maximum height, total biomass, shoot biomass and root biomass (Tables 1 and 2).

**Table 1:** Statistical models for the plant growth during the glasshouse experiment, with as response variables number of leaves, number of shoots, and maximum plant height. The number of leaves and the number of tillers were analyzed with a generalized linear model with negative binomial distribution, and the deviance (Chi‐squared test) is shown for the factors. Maximum plant height is analyzed with an ANOVA, and the *F* values are shown

Factor	*df*	Number of leaves	Number of shoots	Maximum plant height
Species	1	3,130.82***	717.35***	9.55**
Salinity	5	24.82***	24.05.29***	9.07***
Salt Spray	1	35.30***	18.85***	4.77*
Days	1	1,015.39***	66.25***	535.21***
Species × Salinity	5	390.56***	163.92***	11.26***
Species × Salt Spray	1	29.81***	15.14***	5.02*
Species × Days	1	86.61***	110.51***	31.94***
Salinity × Salt Spray	5	132.20***	74.05***	1.11
Salinity × Days	5	22.28***	19.09**	7.25***
Salt spray × Days	1	0.15	0.39	0.075
MS_residuals_	2,074	–	–	188

*df*, degrees of freedom.

The asterisk denotes the level of significance (*p < .05, **p < .005, ***p < .001).

**Table 2:** ANOVA model for the total biomass, shoot/root ratio, shoot biomass, root biomass. The values shown are the *F* values

Factor	*df*	Total biomass	S/R ratio	Shoot biomass	Root biomass
Species	1	96.73***	109.00***	174.39***	15.23***
Salinity	5	36.47***	0.81	7.87***	11.85***
Salt Spray	1	0.39	0.00	0.14	0.48
Species × Salinity	5	35.91***	44.58***	10.89***	9.79***
Species × Salt Spray	1	0.55	1.03	0.79	0.50
Salinity × Salt Spray	5	0.07	0.01	0.68	0.78
MS_residuals_/SS_residuals_	172	5.77	0.12	1.9	1.47

*df*, degrees of freedom.

The asterisk denotes the level of significance (***p < 0.001).

**Figure 3:**
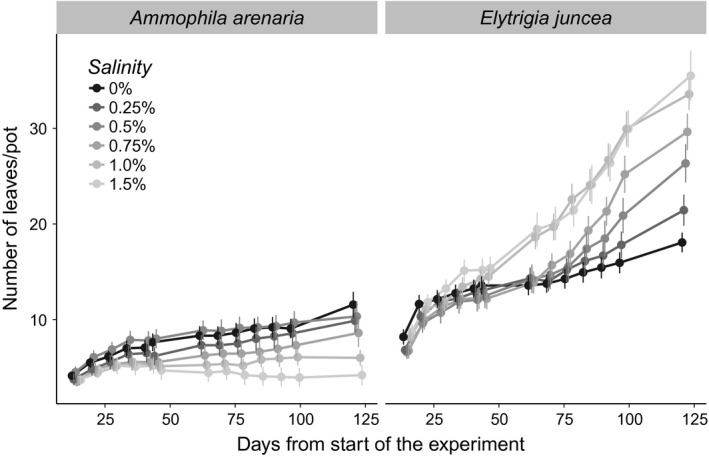
The number of living leaves per pot for *Ammophila arenaria* and *Elytrichia juncea* over the first 125 days of the experiment at the different soil salinity levels. The points are means, and the error bars are standard errors

**Figure 4:**
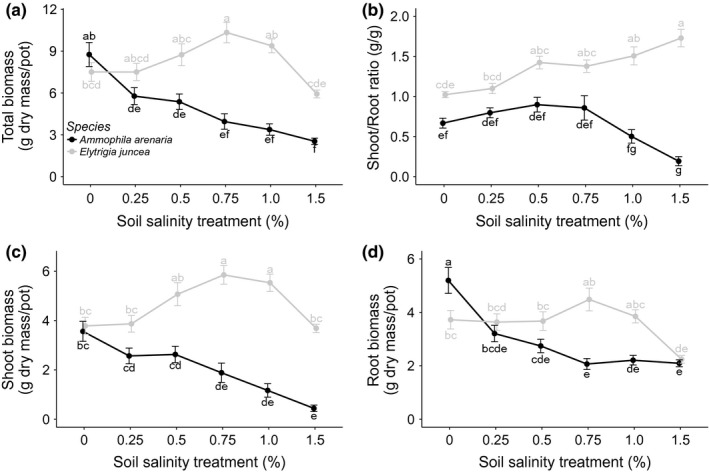
The effect of soil salinity (%) and species (*Ammophila arenaria* and *Elytrichia juncea*) on: (a) total biomass (g dry mass/pot); (b) shoot/root ratio (g/g dry mass); (c) shoot biomass (g dry mass/pot); (d) root biomass (g dry mass/pot). The points are the means, and the error bars are the standard error. The letters denote the significance between the different salinity levels (Tukey's HSD test)

Soil salinity significantly affected plant performance, with the effect strongly depending on species. For *A. arenaria,* we found a significant negative effect of soil salinity on the shoot biomass, root biomass, and total biomass, number of living leaves (Figures 3 and 4), tillers, and maximum height (Tables 1 and 2). Treatment effects on living leaves became significant from 50 days onward (Figure 3). Plant biomass at harvest was negatively related to soil salinity. Biomass decreased by 34% between the 0% and 0.25% treatments. The decrease in biomass was mainly due to the decrease in shoot biomass (Figure 4c). Although the root biomass clearly decreased between 0% and 0.25% salinity, the root biomass did not further decrease at higher soil salinity levels (Figure 4d). Consequently, the decrease in shoot biomass resulted into a lower shoot to root ratio at high soil salinity (1.0%, 1.5%) (Figure 4b). The fraction of dead leaves increased with soil salinity for *A. arenaria* (*F*_1196,5_ = 4.48, *p* < .001), the fraction of dead leaves from 0.17 ± 0.020 alive/dead in the control treatment to 0.39 ± 0.043 alive/dead in the highest soil salinity treatment. Mortality of all *A. arenaria* plants occurred in nine experimental pots (9.4%). Plant mortality for the highest soil salinity treatment was 43.8% (seven of nine pots that had been subjected to the 1.5% soil salinity treatment). The other two experimental pots were with 0.75% soil salinity treatment and 0. 5% soil salinity treatment. Increased soil salinity resulted into smaller and thinner, yet denser leaves, significantly decreasing SLA (*F*_77,5_ = 6.54, *p* = <.001), from 39.58 ± 3.04 cm/g in the control treatment to 13.99 ± 5.70 cm/g in the highest soil salinity treatment. Leaf thickness ranged from 0.41 ± 0.017 mm in the control treatment to 0.18 ± 0.063 mm in the highest salinity treatment (*F*_37,5_ = 5.72, *p* < 0.001). The ratio between dead and total shoot biomass was significantly affected by the soil salinity treatment (*F*_81,5_ = 15.47, *p* = <.001), the ratio between dead and total shoot biomass was 0.08 ± 0.048 g/g for the control treatment and 0.50 ± 0.094 g/g for the highest soil salinity treatment. We found no significant relationship between the dead leaves biomass and Na concentration (*F*_44_ = 1.33, *p* = .25).

**Figure 5:**
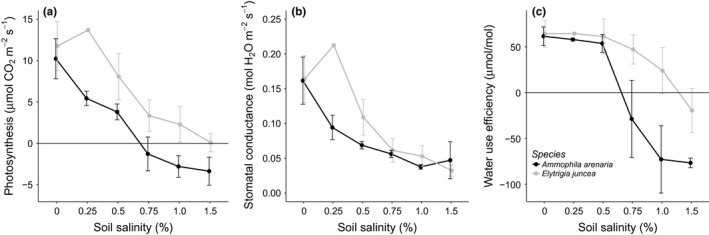
The mean and standard error of (a) the photosynthesis (Asat) (μmol CO_2_ m^−1^ s^−1^), (b) the stomatal conductance (mol H_2_O m^−2^ s^−1^), and (c) the water use efficiency (μmol/mol) for *Ammophila arenaria* and *Elytrichia juncea* at different soil salinity levels. There were no significant differences between the different species and soil salinities

In contrast to *A. arenaria*, increasing soil salinity generally improved performance of *E. juncea*. The number of living leaves, tillers, and maximum plant height increased linearly with soil salinity (Table 1 and Figure 3), and the fraction of dead leaves was higher at low salinity (0.48 ± 0.022 alive/dead) than high salinity (0.26 ± 0.0011 alive/dead, *F*_1227,5_ = 18.01, *p* < .001). Plant biomass at harvest showed an optimum at a soil salinity of 0.75%. At this salinity level, the total biomass was 37.7% higher than for to the 0% salinity treatment (Figure 5a). At the highest soil salinity level (1.5%), the total biomass was about equal to that of the control treatment with 0% soil salinity. The effect of soil salinity on the total biomass of *E. juncea* was mainly driven by the effect on shoot biomass (Figure 4c). The root biomass did not show a significant increase at the soil salinity levels of 0.25%–1.0%, but decreased at 1.5% soil salinity (Figure 4d). Consequently, the shoot to root ratio increased with increasing soil salinity for this species (Figure 4b), again a response opposite to that of *A. arenaria*. Increased soil salinity also resulted in smaller and denser leaves, decreasing the SLA from 93.49 ± 6.56 cm/g in the control treatment to 64.26 ± 3.53 cm/g in the highest soil salinity treatment (*F*_77,5_ = 6.54, *p* = <.001). In contrast to *A. arenaria,* soil salinity did not affect leaf thickness for *E. juncea* (*F*_35,5_ = 2.11, *p* = .087, leaf thickness control treatment: 0.31 ± 0.016, highest salinity treatment: 0.29 ± 0.015). For *E. juncea,* no distinction has been made between the dead and alive biomass; however, from the data collected during the experiment, it seemed that the number of dead leaves was similar to that of *A. arenaria*.

### 3.5 Impacts on photosynthesis

Photosynthesis (Asat) was affected by both soil salinity and species, with salt spray having no effect (Table 3 and Figure 5a). Photosynthesis linearly decreased with soil salinity for both species (Table 3 and Figure 5a), with *A. arenaria* showing a stronger response than *E. juncea*. Photosynthesis of *A. arenaria* was completely suppressed (i.e., there was respiration instead of CO_2_ accumulation, reflected by negative values) at soil salinity levels of 0.75% and higher, whereas of *E. juncea* kept photosynthetically active in all treatments. Stomatal conductance decreased with soil salinity, irrespective of species (Table 3 and Figure 5b). As a result, the WUE of both species decreased with increasing soil salinity, dropping below zero at soil salinity levels of 0.75% or higher for *A. arenaria*, and at a soil salinity of 1.5% for *E. juncea* (Figure 5c).

**Table 3:** An overview of the model outcome of the photosynthesis (NCE), stomatal conductivity and water use efficiency (WUE). The data were analyzed with an ANOVA type 3. The values shown are the *F* values

Factor	*df*	Photosynthesis	Stomatal conductivity	WUE
Species	1	0.017	0.032	0.00
Salinity	5	4.23**	2.81*	2.94*
Salt Spray	1	0.96	0.15	2.85
Species × Salinity	5	0.16	0.28	0.57
Species × Salt Spray	1	1.13	0.0091	0.57
Salinity × Salt Spray	5	0.50	0.38	0.94
SS_residuals_	25	562.42	8.46	20.87

*df*, degrees of freedom; WUE, water use efficiency (μmol/mol).

The asterisk denotes the level of significance (*p < 0.05, **p < 0.005).

### 3.6 Leaf nutrient concentrations

**Figure 6:**
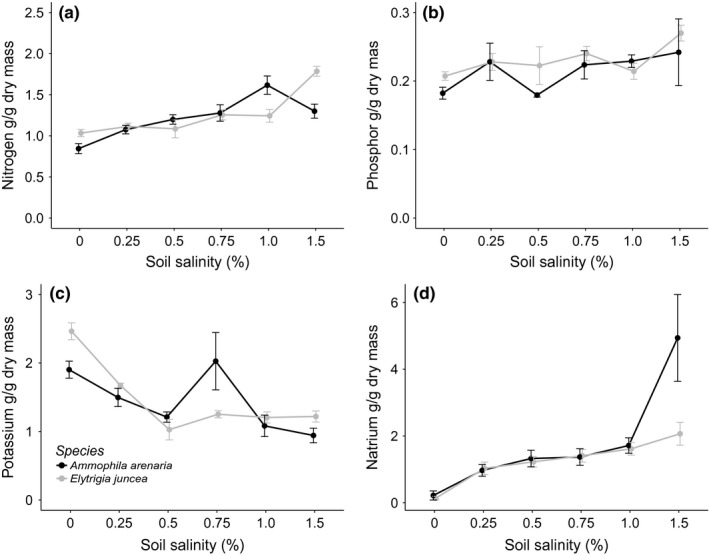
The mean and standard error of (a) N concentration (g/g dry mass), (b) P concentration (g/g dry mass), (c) K concentration (g/g dry mass), and (d) Na concentration (g/g dry mass) of *Ammophila arenaria* and *Elytrichia juncea* for different soil salinity levels. As there was a significant effect of the salt spray treatment on the Na concentration, for graph 6D, we only present data without the salt spray treatment. There were no significant differences between the different species and soil salinities

Leaf nutrient (N, P and K) concentrations were comparable between the two grass species and N and P concentrations increased with soil salinity, while K concentrations decreased with soil salinity (Table 4 and Figure 6a–c). Leaf Na concentrations differed between species, with *A. arenaria* having significantly higher leaf Na concentration compared to *E. juncea*. This species effect was mainly caused by the effect of salt spray on leaf Na concentration: Salt spray increased the leaf Na concentration of *A. arenaria* but did not affect *E. juncea*. Leaf Na concentrations increased with soil salinity for both species at approximately the same rate (Table 4 and Figure 6d). The leaf Na and K concentrations were negatively correlated to each other (*K* = 0.75–0.46*Na, *p* = .009, *R*_2_ = 0.06).

**Table 4:** An overview of the model outcome of the N, P, K^+^, Na^+^ concentrations in the leaves. The data were analyzed with an ANOVA. The values shown are the *F* values

Factor	*df*	N	P	K	Na
Species	1	0.97	2.78	0.039	8.66**
Salinity	5	19.83***	2.92*	16.28***	46.07***
Salt Spray	1	2.88†	0.059	0.79	5.04*
Species × Salinity	5	7.36***	0.65	4.87***	1.92
Species × Salt Spray	1	2.34	0.68	4.80*	3.73†
Salinity × Salt Spray	5	1.17	1.13	2.90*	1.94†
MS_residuals_	74	0.041	0.0026	0.17	0.31

*df*, degrees of freedom.

The asterisk denotes the level of significance (^†^ *p* < 0.1,*p < 0.05, **p < 0.005, ***p < 0.001).

## 4 DISCUSSION

In this study, we tried to explain the discrepancy in the current literature between spatial plant distribution on the beach, salinity‐tolerance ranges measured in short‐term physiological studies and the actual salinity measured on the beach. We hypothesized that the discrepancy was either related to (i) the interactive effects of aboveground salt spray stress and belowground salinity stress or (ii) the short experimental duration of most glasshouse experiments. Contrary to our expectations, we found in our glasshouse experiment that salt spray did not interact with soil salinity. Moreover, despite species being up to three times more sensitive to soil salinity in our 176 day glasshouse experiment than in a similar 32 day glasshouse experiment (Sykes & Wilson, 1989), the concentrations found to limit species performance in the glasshouse were still several orders of magnitude higher than observed in the field. Below, we try to answer the questions to what extent soil salinity restricts the spatial distribution of dune building species, and thereby the development of vegetated dunes, on the beach.

### 4.1 Salt spray

Salt spray did not affect growth of the main dune building species in our glasshouse experiment, also at high soil salinity concentrations. The absence of an effect is unlikely to be an artifact of the concentrations used in our study. Although we did not control the droplet size in our study (Boyce, 1954), the leaf salt concentration that the plants were subjected to in our experiment is likely to exceed the concentrations experienced by plants in the field, because of the absence of precipitation in the glasshouse. In the field, salt stress is often reduced by precipitation, as the rain removes buildup of salt from the leaves (Boyce, 1954). The tolerance for salt spray of species growing on the beach has also been found in other studies (Rozema et al., 1983; Sykes & Wilson, 1988) and has been mainly attributed to the structure of the epicuticular wax layer (Ahmad & Wainwright, 1976), which can reduce the uptake of Na^+^ and Cl^−^ deposited by salt spray.

### 4.2 Mechanisms explaining biomass response to saline conditions

We explored to what extent the species biomass responses to increasing soil salinity in the glasshouse could be attributed to nutrient limitation, osmotic stress, and ionic stress, as this could, perhaps, shed more light on species responses in the field. Of these three mechanisms, nutrient limitation is unlikely. Leaf N and P concentrations remained constant or increased with increasing soil salinity levels. Leaf K concentration did decrease with soil salinity, suggesting K limitation as a potential explanation for a negative biomass response to high salinity. However, as K concentration decreased irrespective of species, and leaf K concentrations remained within field ranges (1%), K deficiency as driver of the biomass response seems unlikely. The decline in K was positively related to the increase in leaf Na concentration, presumably due to the competition between K and N uptake at the root surface (Amtmann & Sanders, 1998; Colmer & Flowers, 2008). This leaves osmotic stress and ionic stress as alternatives to explain the negative biomass responses. Soil salinity stress occurs in two stages for a plant (Munns & Tester, 2008): first, a rapid response to increase in external osmotic pressure (osmotic stress), and second, over time a response to the accumulation of Na^+^ in the leaves (ionic stress) (Munns & Termaat, 1986). Osmotic stress characteristically results in the reduction in shoot growth (Weimberg, Lerner, & Poljakoff‐Mayber, 1984), while ionic stress leads to leaf mortality. Very likely both processes played a role, with osmotic stress being important for both *A. arenaria* and *E. juncea*, while ionic stress may have contributed to the biomass response of *A. arenaria* only.

For both species, stomatal conductance decreased with increasing soil salinity, the decrease being steeper for *A. arenaria* than for *E. juncea*. The reduction in stomatal conductance, which indicates stomatal closure, is often associated with osmotic stress (Lovelock & Ball, 2002; Munns, 1993). For *A. arenaria,* the reduction in leaf stomatal conductance was accompanied by a steep decline in leaf photosynthesis for soil salinities until 0.75%. The decline in photosynthesis mirrored the pattern observed for shoot biomass, suggesting the species increasingly suffered from osmotic stress. For *E. juncea*, photosynthesis declined less steeply, remaining positive until the highest soil salinity treatment of 1.5%. Surprisingly, the physiological response did not mirror the response in shoot biomass, which showed an optimum at 0.75% soil salinity. The above suggests that *E. juncea* also experienced increasing osmotic stress with increasing soil salinity, but was better able to compensate for it than *A. arenaria*. The reason for the discrepancy between the biomass and photosynthetic responses is as yet unclear and could perhaps be related to a time‐lag effect between photosynthetic rates and biomass production suggesting cumulative stress.

Ionic stress is caused by the Na^+^ accumulation in the leaves until toxic levels are reached, causing senescence (Munns & Termaat, 1986; Munns & Tester, 2008). Both *A. arenaria* and *E. juncea* showed a similar increase in the Na concentration in the leaves, but only *A. arenaria* displayed increasing leaf mortality with increasing soil salinity. For *E. juncea,* however, the proportion of dead leaves was higher at low salinity levels. The above suggests that for the soil salinity range we studied, ionic stress may have been an issue for *A. arenaria*, but not for *E. juncea*. Perhaps *E. juncea* could be more tolerant to Na by storing it in different cell organs, for example, the vacuole (Flowers, Troke, & Yeo, 1977).

### 4.3 Distribution and growth in the field

Vegetation distribution on the beach has often been hypothesized to depend on soil salinity (Westhoff et al., 1970), as the species zonation corresponds to different degrees in salinity tolerance of dune building species investigated under controlled conditions. Our results from the field transplantation experiment suggest that there is an abiotic factor restraining plant survival in winter, but not in summer. In summer, the growth of *A. arenaria* in zone II was limited by soil salinity, but not that of *E. juncea*. *Elytrigia juncea* showed no significant difference in plant growth in the field between the three different zones with different soil salinity. Unfortunately, we do not have any results on plant performance of transplants in zone I due to anthropogenic disturbance. Nevertheless, as soil salinity in the unvegetated zone I was comparable to *E. juncea* occupied zone II, it seems reasonable to assume that a factor other than salt stress prevented vegetation development in this zone. Perhaps more regular inundation by the sea in zone I and associated mechanical stress or, alternatively, higher extremes in soil salinity could explain the pattern.

In our study, we measured salt concentrations at one moment in time, whereas soil salinity is known to vary extensively in the field, due to changes in sea level, salt spray, and precipitation (de Jong, 1979; Maun, 2009). Of course, it is possible that plant distributions reflect higher salt concentrations that are reached after periodic drought in summer (Ayyad, 1973), or perhaps, directly after storm inundation in winter (Barbour & DeJong, 1977). Furthermore our results are only based on the Hors, Texel, and the soil salinity could be different at other beaches. Yet, despite the limitation of our study described above, soil salinity at two other beaches differing in morphology was even lower than the level we found on the Hors, Texel. This is also consistent with the few other studies that measured soil salinity on the beach, which reported soil salinity levels between 0.0008% and 0.04% (Boyce, 1954; de Jong, 1979; Gooding, 1947; Kearney, 1904; Olsson‐Seffer, 1909). At these beaches where soil salinity remains well below 0.25%, the dune development of *A. arenaria* is probably not limited by soil salinity. In general, beach soil salinity probably depends on beach morphology. Shorter beaches, with a steep slope, and a higher elevation might have lower soil salinity levels compared to wide beaches, with a gradual slope and a lower elevation. Further research should be conducted on the variation of soil salinity on the beach and its relationship to beach morphology and extreme events, such as drought and inundation.

Our field experiment suggests that the survival of dune building grasses is determined in the winter season. In zone II, no plants of either species survived the winter. The low survival of both species is most likely associated with the occurrence of a storm that winter, which resulted in high water levels. Storms can severely erode dunes (Claudino‐Sales, Wang, & Horwitz, 2008; Haerens, Bolle, Trouw, & Houthuys, 2012; Keijsers, Poortinga, Riksen, & Maroulis, 2014) and have been found to be a limiting factor for embryo dune development (van Puijenbroek, Limpens, et al., 2017). The storm affected probably all experimental plots; however, the zones closer to the sea were probably longer and/or more deeply inundated by high water compared to zones further from the sea (Barbour & DeJong, 1977). Even though the transplanted plants did not survive the winter period in our field experiment, natural *E. juncea* dunes do occur in zone II. Why our transplanted plants did not survive while the natural plants did, we cannot say for sure. It is possible that the better developed root system, the higher cover, and/or bigger dune size of the natural plants increased their storm resistance.

### 4.4 (Dis)similarities between field and glasshouse

The plant species differed in vigor between glasshouse and field: In the field, *A. arenaria* grew much better than *E. juncea* at the same soil salinity than in the glasshouse experiment.

This difference between the glasshouse and field experiment is most likely caused by factors that are important for species growing in the field, but were not included in the glasshouse experiment, such as sand burial, precipitation, and storm erosion. With sand burial, *A. arenaria* can escape soil pathogens, which promotes the growth of *A. arenaria* (Maun, 1998; van der Putten, 1989). Although we used sterile river sand for our glasshouse experiment, pathogens could have been introduced with the rhizomes which we collected in the field (de Rooij‐van der Goes, Peters, & van der Putten, 1998). Furthermore sand accumulation might decrease the soil salinity by increasing elevation. *Elytrigia juncea* is not known to suffer from negative soil feedback in the field but can suffer from the high rates of sand burial during winter, particularly in zones close to the dunes, such as zone IV (Sykes & Wilson, 1990). In the field, both species trapped sand; however, the amount was not much, between 10 and 20 cm in elevation change, and did not differ much between the species.

In the field, *A. arenaria* decreased in the number of leaves in response to increasing soil salinity over summer; however, the decrease was not so pronounced as in the glasshouse given equal salinity. Perhaps this difference can be explained by the temporary dilution of soil salinity, and thus alleviation of salt stress, by precipitation in the field (Greaver & Sternberg, 2007; Seeliger, Cordazzo, Oliveira, & Seeliger, 2000).

In the field, both species declined dramatically in performance over winter. This decrease was probably caused by a large storm that occurred during our study period. Storms have two main effects, they cause mechanical erosion of the dunes and they increase the salinity in the soil by seawater inundation (Charbonneau, Wootton, Wnek, Langley, & Posner, 2017; Feagin et al., 2015; Sigren, Figlus, & Armitage, 2014). Seawater has a high salinity of 3.5% that could have a detrimental effect on the growth of both *A. arenaria* and *E. juncea* (Konlechner et al., 2013). However, the inundation of seawater by storms mainly occurs during the winter season, and it is not clear how detrimental this increased salinity is for plants when they are not growing. A worthwhile avenue for future research is to study the effect of inundation and resulting increase in soil salinity on the survival and growth of dune building grasses during the growing season and winter season. In zone III, *E. juncea* had a lower survival compared to *A. arenaria,* whereas the results from our glasshouse experiment suggest that *E. juncea* is more resistant to soil salinity than *A. arenaria*. Consequently, the low survival of *E. juncea* and *A. arenaria* is most likely caused by the mechanical erosion of the dunes and vegetation. The higher survival of *A. arenaria* compared to *E. juncea* in zone III is most likely due to the higher vegetation density of *A. arenaria*, which enables the species to better withstand mechanical erosion by storms. However, the higher vegetation density was partly the result of a lower productivity of *E. juncea* compared to *A. arenaria*. From this study, it is difficult to predict whether the differences in winter survival of *A. arenaria* and *E. juncea* would be similar if they had equal number of leaves. However as *E. juncea* had a lower growth rate compared to *A. arenaria* in the field, our results still suggest that mechanical erosion by storms is more likely to limit the distribution of *E. juncea* than soil salinity; however, we cannot totally excluded the effect of episodic increase in soil salinity during storms.

### 4.5 Implication for dune development

Although some research has been conducted on factors that determine plant succession in dunes, research on factors that determine the vegetation limit on the beach is scarce. *Ammophila arenaria* has been introduced in many countries for its dune building capabilities; therefore, understanding the factors that determine its vegetation limits would be beneficial. Dune development starts with the establishment of vegetation on the beach making it dependent on establishment of dune building species from rhizome or seeds (Harris & Davy, 1986; Hilton & Konlechner, 2011; Maun, 1981). Although glasshouse results show clear differences in salt tolerance between both dune building species, beach salinity and performance of transplanted species on the beach suggest that salt stress is unlikely to drive species distribution or limit dune building on the beaches we studied. We cannot exclude however that on some beaches salt salinity does affect distribution of *A. arenaria,* provided soil salinity on the beach reaches concentrations above 0.25%. Especially, the high soil salinity could prevent the germination of *A. arenaria* seeds, as seedlings are more vulnerable to soil salinity (Sykes & Wilson, 1989). A limited germination of *A. arenaria* could explain why only *E. juncea* dunes occur in zone II. In contrast to *A. arenaria*, distribution of *E. juncea* is unlikely to be limited by salt stress as illustrated by its natural distribution that shows the species can establish and survive in zone II, which had equal salinity to zone I. As both species facilitate dune development, our results suggest that net dune building on the beach is not limited by soil salinity.

Instead of soil salinity, dune development seems more limited by the storms in the winter season. Storms during the winter season result in mechanical erosion, where vegetation can be completely removed by waves. The sensitivity to mechanical erosion could differ between dune building species, however, with denser vegetated species being less sensitive to mechanical erosion, than species forming a more sparse vegetation (Charbonneau et al., 2017; van Puijenbroek, Nolet, et al., 2017). Sensitivity to mechanical erosion also depends on the root network, although this has hitherto not been investigated for coastal dunes (Feagin et al., 2015). Both of the species studied expand with rhizomes, and as a result, most shoots are connected to each other. The rhizome network likely promotes stabilization of sediment and reduces storm erosion, potentially increasing survival over winter. In our field experiment, we planted individual plants which might have made them more sensitive to mechanical erosion that vegetated dunes in the field. Rhizomes are also known to be quite resistant against high soil salinity, and rhizomes have been found to be viable after floating 70 days in seawater (Konlechner & Hilton, 2013). Therefore, storm erosion could result in the mortality of the shoots, but next growing season vegetation growth might occur from the rhizomes. Taken together, our results suggest that there is no fixed vegetation limit on the beach, but rather a combination of continuous summer recruitment and stochastic winter mortality, with net expansion of dune building species and dunes depending on storm characteristics of the winter season. The limits for vegetation establishment on the beach are important for modeling coastal dune development (de Groot et al., 2012; Durán & Moore, 2013).

## 5 CONCLUSION

The purpose of this study was to assess to what extent soil salinity restricts the spatial distribution of dune building species, and thereby lower the limit for development of vegetated dunes on the beach. Performance of dune building species did change with soil salinity in the glasshouse, confirming salt stress as a potential limit for vegetation growth, but field measurement on plant performance and summer soil salinity suggest that mortality of dune building grasses is rather a function of mechanical erosion in winter, rather than summer soil salinity. Consequently, our findings suggest that soil salinity stress either restricts recruitment from seeds, takes place in winter, or that development of vegetated dunes is less sensitive to soil salinity than hitherto expected.

## CONFLICT OF INTEREST

None declared.

## AUTHOR CONTRIBUTIONS

MvP, JL, CT, and NM conceived the idea; MvP, CT, and NM set up the experiment and collected the data; IO, MvP, and JL collected the physiological data; MvP, JL, and FB analyzed the data; All authors contributed critically to the drafts and gave final approval for publication.

## DATA ACCESSIBILITY

Data supporting the results are archived in the 4TU Datacentre https://data.4tu.nl/, under https://doi.org/10.4121/uuid:01561d16-bc87-4614-8f4e-e17393d4d34d.
