# Supplementary material for: Does salt stress constrain spatial distribution of dune building grasses Ammophila arenaria and Elytrichia juncea on the beach?
Source: Ecol Evol. 2017 Aug 8;7(18):7290–303. doi: 10.1002/ece3.3244 (PMC5606859; doi:10.1002/ece3.3244)
Supplement: Supplementary file 1 [file ECE3-7-7290-s001.docx]

**Supplementary data S1**

Overview table with literature on the response of *A. arenaria* and *E. juncea* on salt spray and soil salinity.

**Table S1.1** Overview table with literature on the response of *A. arenaria* and *E. juncea* on salt spray and soil salinity. The treatment indicates the abiotic conditions the authors tested experimentally.

| Authors | **Treatment** | **Method** | **Species** | **Results** |
| --- | --- | --- | --- | --- |
| Salt Spray | | | | |
| Rozema et al, 1983 | Distilled water and seawater | Once every two days  Experiment duration: 2.5 months | *E. juncea* | No effect of salt spray on biomass, only slight increase of Cl^-^ concentration in the leaves |
| Sykes & Wilson, 1988 | Distilled water and 3.5% NaCl | Once every 5 days  Experiment duration: 21 days | *A. arenaria* | No effect on biomass, seedling were affected. |
|  |  |  | *E. juncea* | No effect on biomass |
| Soil salinity | | | | |
| Rozema et al 1983 | 0 mM NaCl = 0%  60 mM NaCl = 0.35 %  150mM NaCl= 0.87%  300mM NaCl = 1.74% | Aerated hydroculture  Experiment duration: 6 weeks | *E. juncea* | Overall decrease in growth. Decrease in K^+^ concentration in the leaves, increase in Na^+^ and chloride concentration in the leaves. |
| Sykes & Wilson 1989 | 0%, 0.25%, 0.5%, 0.75%, 1%, 2% | Pots were drained  Experiment duration: 32 days | *A. arenaria* | Significant decline with higher soil salinities, RGR significantly different at 0.75% soil salinity |
|  |  |  | *E. juncea* | Shows an optimum at 0.75% soil salinity, no overall significant difference |
| Konlechner et al 2013 | 3.5% sea water | Immersed 3 hours  Growth assessed for a week | *A. arenaria* | No survival when immersed with seawater, some plants survived if they were washed with fresh water. |

**Supplementary data S2**

**
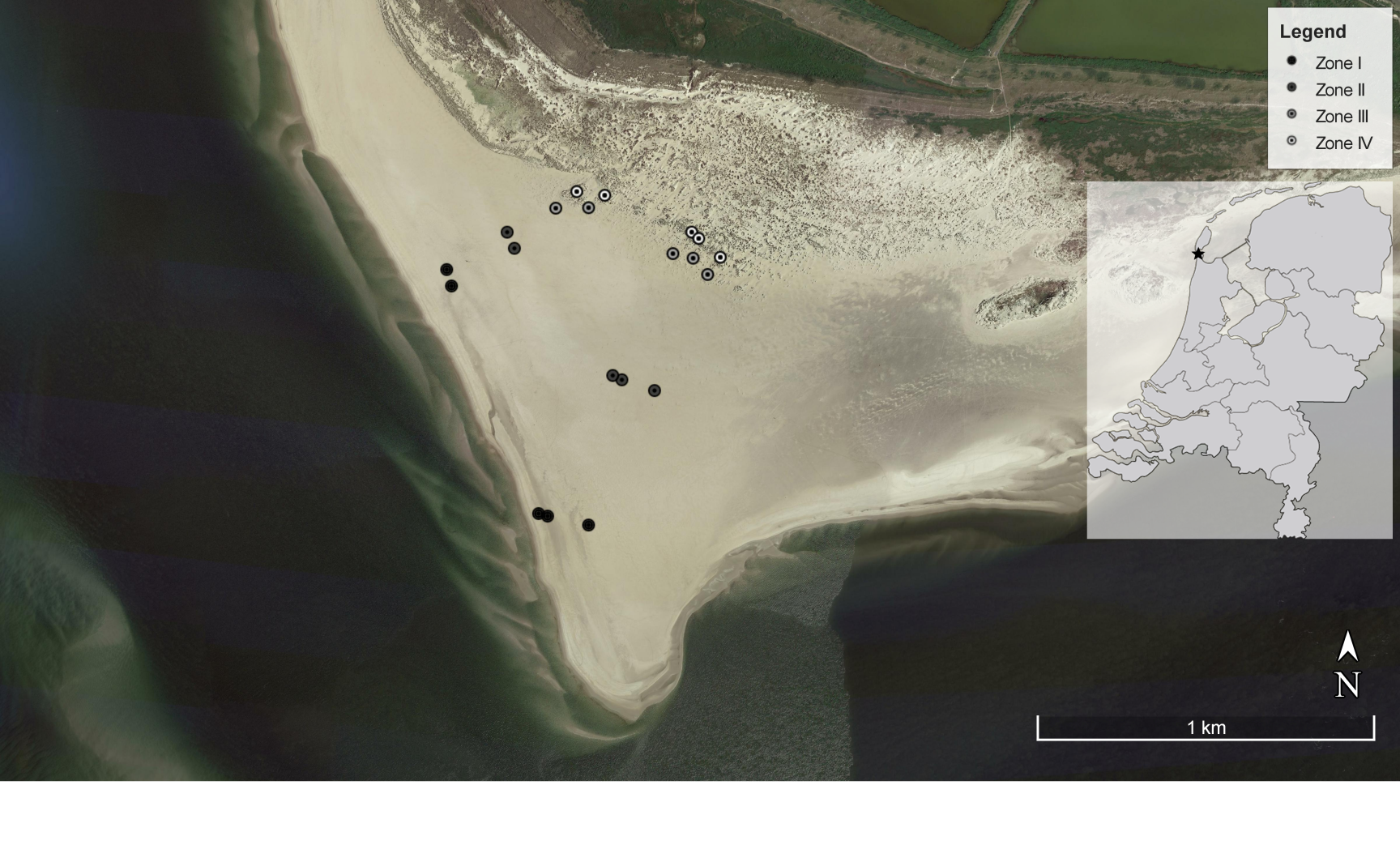
**Overview of the field site Hors at Texel.

**Figure S2.1** Overview of the field site on the Hors at Texel. The dots indicate the different zones were we conducted the field transplantation experiment and measured the soil salinity. The star in the map indicates the location of the field site in the Netherlands. Zone I is the non-vegetated zone , zone II the zone with only *E. juncea* occurring, zone III the zone with both *E. juncea* and *A. arenaria,* and in zone IV *A. arenaria* is dominant.

**Supplementary data S3**

Plant growth of mono and mixed culture in the field experiment

**
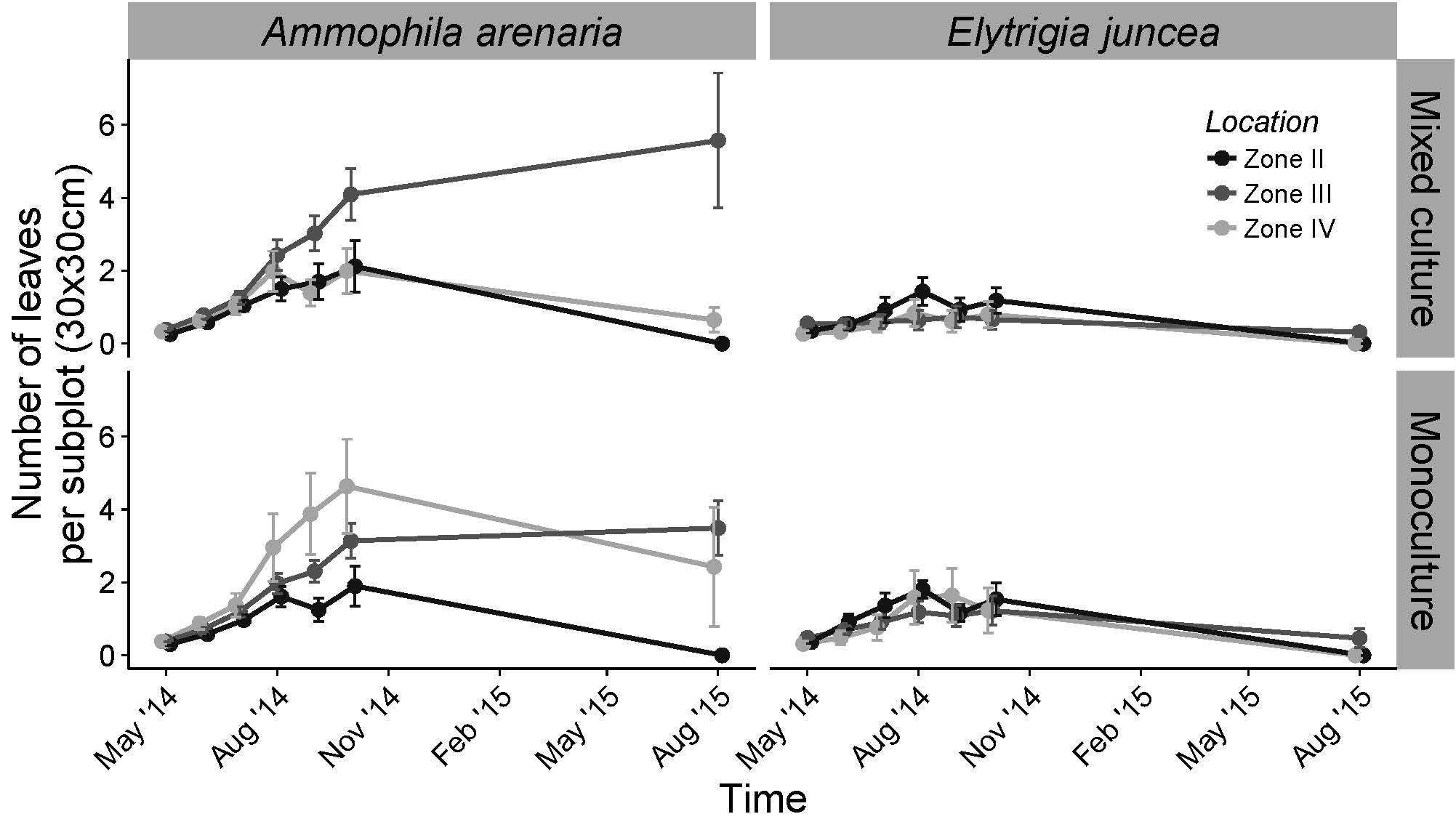
**

**Figure S3.1** The number of living leaves/plant for *A. arenaria* and *E. juncea* and mixed and mono culture per subplot of 30cm x 30cm within a plot at the different zones at the Hors, Texel, over a period of 15 months. The points are means and the error bars are standard errors. Zone II is the zone with only *E. juncea* occurring, zone III the zone with both *E. juncea* and *A. arenaria* and in zone IV *A. arenaria* is dominant.

**Supplementary data S4**

**
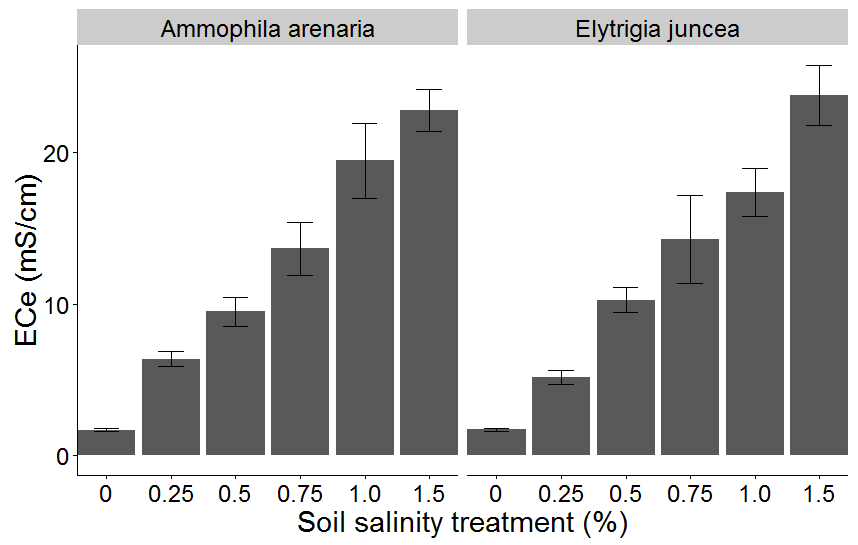
**The ECe (mS/cm) of the experimental plots.

**Figure S4.1** The mean ± SE ECe (mS/cm) for the different soil salinity treatments for both *A. arenaria* and *E. juncea*. The saline solutions that were applied to the pots were: 0% = 0.28 mS/cm, 0.25% = 6.0 mS/cm, 0.5% = 11.1 mS/cm, 0.75% = 16.2 mS/cm, 1.0% = 20.2 mS/cm, 1.5% = 33.9 mS/cm.
